# Bioaccumulation of Non-Essential Trace Elements Detected in Women’s Follicular Fluid, Urine, and Plasma Is Associated with Poor Reproductive Outcomes following Single Euploid Embryo Transfer: A Pilot Study

**DOI:** 10.3390/ijms241713147

**Published:** 2023-08-24

**Authors:** Andrea Palomar, Roberto Gonzalez-Martin, Alicia Quiñonero, Nuria Pellicer, Rocio Fernandez-Saavedra, Isabel Rucandio, Rodolfo Fernandez-Martinez, Estefania Conde-Vilda, Alberto J. Quejido, Caroline Zuckerman, Christine Whitehead, Richard T. Scott, Francisco Dominguez

**Affiliations:** 1 Reproductive Biology and Bioengineering in Human Reproduction, IVIRMA Global Research Alliance IVI Foundation—Health Research Institute La Fe (IIS La Fe), 46026 Valencia, Spain; andrea.palomar@ivirma.com (A.P.); roberto.gonzalez@ivirma.com (R.G.-M.); alicia.quinonero@ivirma.com (A.Q.); nuria.pellicer@ivirma.com (N.P.); 2 Chemistry Division, Department of Technology, Research Centre for Energy, Environment and Technology (CIEMAT), 28040 Madrid, Spain; rocio.fernandez@ciemat.es (R.F.-S.); isabel.rucandio@ciemat.es (I.R.); rodolfo.fernandez@ciemat.es (R.F.-M.); estefania.conde@ciemat.es (E.C.-V.); alberto.quejido@ciemat.es (A.J.Q.); 3 Department of Clinical Research, IVIRMA Global Research Alliance IVI-RMA New Jersey, Basking Ridge, NJ 07920, USA; czuckerman@ivirma.com (C.Z.); cwhitehead@ivirma.com (C.W.);; 4 Sidney Kimmel College of Medicine, Thomas Jefferson University, Philadelphia, PA 19107, USA

**Keywords:** non-essential trace elements, biofluids, infertility, ovarian response, IVF outcomes, live birth

## Abstract

This study aims to determine the association of non-essential trace elements present in follicular fluid, plasma, and urine with reproductive outcomes of women undergoing intracytoplasmic sperm injection (ICSI), preimplantation genetic testing for aneuploidies (PGT-A) and single frozen euploid embryo transfer (SET/FET). This single-center, prospective cohort study included sixty women undergoing ICSI with PGT-A and SET/FET between 2018 and 2019. Urine, plasma and follicular fluid samples were collected on the vaginal oocyte retrieval day to simultaneously quantify ten non-essential trace elements (i.e., Ba, Sr, Rb, Sn, Ti, Pb, Cd, Hg, Sb, and As). We found several associations between the levels of these non-essential trace elements and clinical IVF parameters. Specifically, the increased levels of barium in follicular fluid were negatively associated with ovarian function, pre-implantation development and embryo euploidy, while elevated strontium concentrations in this biofluid were negatively associated with impaired blastulation and embryo euploidy. Elevated plasma strontium levels were negatively associated with ovarian function, fertilization and blastulation. Enhanced presence of other trace elements in plasma (i.e., rubidium and arsenic) were associated with a diminished ovarian function and limited the number of recovered oocytes, mature oocytes and zygotes, respectively. Fully adjusted models suggested significantly lower odds of achieving a live birth when increased concentrations of barium and tin were found in urine.

## 1. Introduction

Non-essential trace elements, including barium (Ba), strontium (Sr), rubidium (Rb), tin (Sn), titanium (Ti), lead (Pb), cadmium (Cd), mercury (Hg), antimonium (Sb), and arsenic (As) are ubiquitous and bioaccumulate in living organisms [1]. Industrialization, fossil fuel energy, and improvements in the agricultural industry have driven increased exposure to these elements [1,2] via occupational exposure and/or intake of industrial pollutants through diet or drinking water [3,4,5]. Exposure to these elements is particularly concerning due to their persistence in plasma and urine, unknown cumulative effects, and potency in human tissues—even slight overexposures have caused serious health complications, including fertility-related issues in animal models [6,7,8]. Further, bioaccumulation of these elements in the follicular fluid can potentially compromise reproductive function [9,10,11,12,13,14]. Even though the mechanisms of toxicity vary between heavy metals, evidence suggests the damage is driven by oxidative stress [15,16]. 

Non-essential trace elements are generally associated with adverse in vitro fertilization (IVF) outcomes [17,18]. Despite mercury not being correlated with the estradiol (E2) concentration on trigger day, increased mercury levels in hair were associated with impaired ovarian reserve, decreased follicle and oocyte yield after controlled ovarian stimulation (COS) [19], and compromised oocyte maturation [20] in IVF patients. In contrast, no associations were found between arsenic and vanadium concentrations in different biological matrices and the number of mature oocytes recovered following COS or between arsenic and barium levels and antral follicle count [9,20,21,22,23,24]. Limited and controversial evidence exists regarding the effect of barium in women undergoing IVF. Studies have reported negative [21] and positive associations [24] with the number of oocytes recovered. Further, the negative association between barium concentration and embryo fertilization rate is reflected by the increased risk of embryo arrest [21] and aligns with the compromised embryo quality observed after lead and cadmium exposure [25]. Besides no effects of barium on IVF outcomes having been reported, positive associations between thallium or mercury exposure and blastocyst rates have been described [24].

Increased levels of mercury, cadmium, and lead exerted moderate effects on clinical IVF outcomes such as implantation [12,22,25,26], clinical pregnancy [12,22,23,27], and live birth rate (LBR) [23,26] inconsistently across studies [12,20,23,24,25,26,27,28]. While no associations have been described between exposure to arsenic, nickel, vanadium, barium, or thallium and the likelihood of clinical pregnancy [20,22,23,24], a greater risk of miscarriage was positively associated with exposure to heavy metals [25], and mercury is postulated to not only delay the time to pregnancy, but also increase the risk of abortion [28]. Taken together, this evidence highlights a need to clarify the extent to which exposure to metals affects reproductive success.

Based on these findings, we reasoned that non-essential trace elements imbalances in women undergoing fertility treatments could threaten their likelihood of reproductive success. Thus, the aim of this study was to delve deeper into the relationships between IVF outcomes and a broad spectrum of non-essential trace elements (simultaneously measured in follicular fluid, plasma, and urine) in women undergoing IVF with preimplantation genetic testing for aneuploidies (PGT-A) and single frozen embryo transfer (SET/FET), to avoid the bias and potential contribution(s) of embryo aneuploidies to reproductive failure.

## 2. Results

### 2.1. Baseline Demographics and Reproductive Characteristics

Within this population, the median age was 33.40 years (IQR 31.37–36.50), the median BMI was 23.87 kg/m^2^ (IQR 21.57–26.30), and the median AMH concentration was 3.60 ng/mL (IQR 2.49–5.17). Most of the study population was Caucasian (71.7%) and 81.7% of the recruited patients had never smoked (Table 1). During COS, patients received a total of 2100.00 IU of follicle-stimulating hormone (FSH; IQR 1800.00–2700.00) and 1125.00 IU of luteinizing hormone (LH; IQR 675.00–1443.75). The median serum E2 level on trigger day was 3750.65 pg/mL (IQR 2622.20–5204.62).

A median of 17.00 oocytes per patient was recovered (IQR 11.00–24.25), and 77.47 ± 14.30% of recovered oocytes were mature. Fertilization, blastulation, and euploidy rates were, respectively, 81.44 ± 16.29%, 55.62 ± 21.47%, and 60.17 ± 23.72%. Among the 55/60 (91.7%) participants who underwent SET/FET, the implantation rate was 80.0%, while clinical pregnancy and live birth rates were 69.1% and 63.6%, respectively. Ultimately, 58.3% of the patients achieved their reproductive goal (Table 1 and Table S1).

### 2.2. Correlations of Non-Essential Trace Elements between and within Biological Matrices

The trace elements’ distributions, geometric mean, and detectable levels in follicular fluid, urine, and plasma on the day of vaginal oocyte retrieval (VOR) are presented in Table 2. Overall, Ba, Sr, Rb, Hg, Ti, and Pb were detected in most of the samples, whereas As could only be quantified in 15.4% of follicular fluid samples. Notably, Sn and Cs were indetectable in follicular fluid, and Sb could not be detected in any of the three biological matrices (Table 2 and Table S2).

**Figure 1:**
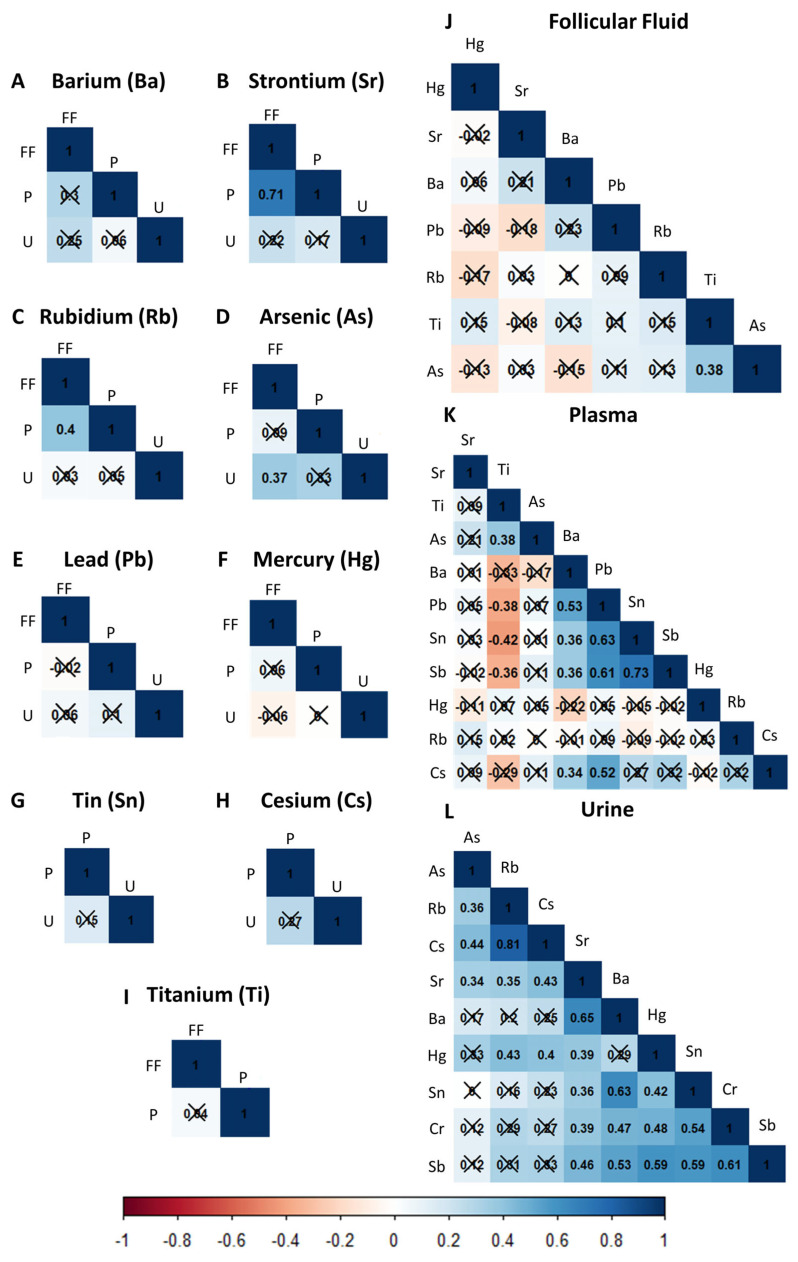
Relationships of ten non-essential trace elements between and within follicular fluid, plasma, and urine. (**A**–**I**) Correlation matrices representing the relationships of the non-essential trace elements between biofluids. (**J**–**L**) Correlation matrices representing the relationships of the elements within each biofluid. FF, follicular fluid; P, plasma; and U, urine. Blue represents a positive correlation, while red represents a negative correlation. Color intensity is proportional to the correlation strength. Strikethrough values represent non-significant associations (*p* > 0.05).

The correlations between the biological matrices for each of the ten non-essential trace elements are displayed in Figure 1A–I. No significant correlations between levels of Ba, Sn, Cs, and Hg in follicular fluid, plasma, or urine were found. However, a strong positive correlation (r = 0.71) between Sr levels in the follicular fluid and plasma was observed. A moderate positive correlation (r = 0.37) was evidenced between the As in follicular fluid and urine. Similarly, we only found a moderate correlation (r = 0.4) between Rb levels in follicular fluid and plasma.

When analyzing each biofluid independently (Figure 1J–L), the strongest positive correlations were found in urine samples. Specifically, urinary Ba levels correlated with urinary Cr (r = 0.47), Sn (0.63), and Sr (0.43). Urinary Cr levels were positively correlated with urinary Sn (r = 0.54), Sr (r = 0.39), Hg (r = 0.48), and Sb (0.61). Sn also had a moderate-high correlation with Sb (r = 0.59), while Sr moderately correlated with Hg (r = 0.39), Sb (r = 0.46), and Rb (r = 0.35). Urinary Hg levels correlated with urinary Sb (r = 0.59) and Rb (r = 0.43) levels. Interestingly, the highest correlation was found between urinary Rb and Cs (r = 0.81), while As had insignificant or low correlations with the other elements. In plasma, Sn and Sb were significantly correlated (r = 0.73), and moderate to strong correlations were observed between Pb and Sn (r = 0.63), Sb (r = 0.61), and Cs (r = 0.52). Interestingly, plasma Ti levels correlated negatively with plasma Sn (r = −0.42) and Pb (r = −0.38) and had the exact same magnitude of correlation (0.38) with As in plasma and follicular fluid. Besides the latter, there were no other significant correlations between trace elements in follicular fluid.

### 2.3. Non-Essential Trace Elements Are Inversely Correlated with Ovarian Response and Preimplantation IVF Outcomes

Following multivariate adjustment for age, BMI, race/ethnicity, and smoking status, the mean differences (95% confidence interval (CI)) in reproductive outcomes were evaluated with respect to the increase between the 20th and 80th percentiles of non-essential trace element concentrations (Table 3 and Table S3).

Several significant negative associations were found between the concentration of non-essential trace elements in the IVF patients’ biofluids and ovarian, fertilization, and embryo development outcomes. Particularly, increased levels of Ba in follicular fluid were inversely associated with serum E2 on trigger day (0.83 [0.70, 0.97]; *p* trend = 0.019), the number of recovered oocytes (0.81 [0.66, 0.99]; *p* trend = 0.042), MII oocytes (0.81 [0.66, 0.99]; *p* trend = 0.041), fertilized embryos (0.79 [0.64, 0.99]; *p* trend = 0.038), blastocysts (0.78 [0.63, 0.97]; *p* trend = 0.027), and euploid embryos (0.73 [0.60, 0.90]; *p* trend = 0.004). Similarly, elevated Sr levels in plasma were significantly associated with lower AMH levels (0.37 [0.15, 0.91]; *p* trend = 0.032), reduced oocyte maturation (0.77 [0.62, 0.96]; *p* trend = 0.022), fertilization (0.77 [0.61, 0.98]; *p* trend = 0.035), and blastocyst development (0.77 [0.60, 0.98]; *p* trend = 0.036). However, elevated Sr concentrations in follicular fluid were only significantly associated with fewer blastocysts (0.76 [0.62, 0.94], *p* trend = 0.014) and euploid embryos (0.77 [0.63, 0.93]; *p* trend = 0.008). Plasma As was also inversely correlated with the number of recovered oocytes (0.74 [0.59, 0.92]; *p* trend = 0. 009), oocyte maturation (0.75 [0.61, 0.92]; *p* trend = 0.008), and the number of fertilized embryos (0.77 [0.62, 0.97]; *p* trend = 0.028). Finally, plasma Rb levels were negatively associated with serum AMH (0.25 [0.08, 0.81]; *p* trend = 0.022).

### 2.4. Elevated Urinary Barium and Tin Reduce the Odds of a Live Birth following Single Frozen Embryo Transfer

**Figure 2:**
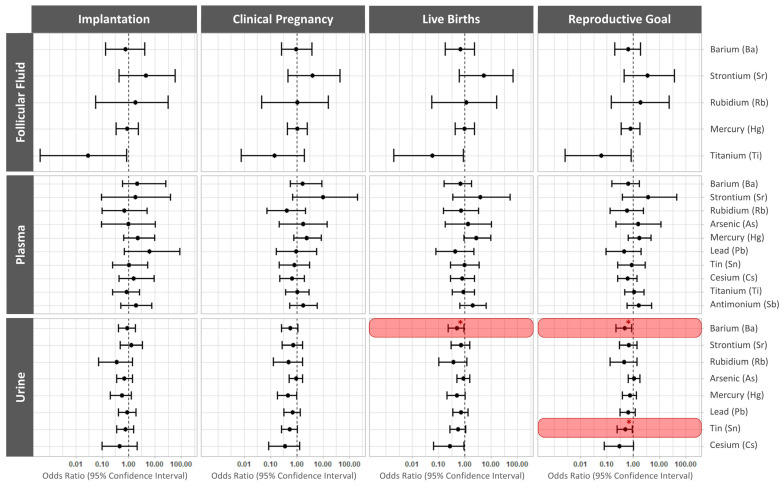
Odds ratios (95% confidence interval) for reproductive outcomes following ICSI and SET/FET. Forest plots present the odds ratios (95% confidence interval) for implantation, clinical pregnancy, live birth per single frozen embryo transfer (SET/FET), and reproductive goal (achievement of a live birth per initiated IVF cycle) across the detectable essential trace elements in each biofluid. * *p* < 0.05. Significant odds ratios are highlighted in red.

The associations between the ten non-essential trace elements present in biofluids on the day of VOR and implantation, clinical pregnancy, live birth rates per transfer, and the reproductive goal are displayed in Figure 2, and Table S4.

In fully adjusted models, increased levels of urinary Ba significantly lowered the odds of a live birth (OR 0.48; 95% CI: 0.22, 0.92; *p* value = 0.04). However, the odds of achieving a live birth with a given cycle were reduced by both elevated urinary Ba (OR 0.49; 95% CI: 0.23, 0.91; *p* value = 0.0036) and elevated urinary Sn (OR 0.5; 95% CI: 0.25, 0.95; *p* value = 0.042). No other non-essential trace element significantly affected reproductive outcomes (Figure S2, Table S4).

## 3. Discussion

This study prospectively evaluated the impact of ten non-essential elements on IVF and reproductive outcomes in a cohort of sixty women undergoing PGT-A and SET/FET. Associations between levels of these elements in follicular fluid, plasma, and urine, collected on VOR day, with various clinical parameters, were analyzed. Since strontium, barium, rubidium, tin, cesium, and antimony were not previously assessed within this context, the current research unveiled novel adverse effects impacting human reproduction.

The impacts of non-essential trace elements on human physiology remain unknown, but slight overexposures to these elements are postulated to cause adverse events. Further, there is rising concern that continued exposure to non-essential trace elements from multiple sources may lead to their bioaccumulation and use as substitutes for essential elements. The presence of the elements described herein may also generate free radicals that cause oxidative damage to proteins and DNA or alter various signaling cascades by modifying gene expression and epigenetic modulation [1,2]. Despite their ubiquitous presence in dietary sources, the environment, and industry, epidemiological studies addressing reproductive risks remain scarce.

Barium is mainly bioaccumulated in the human body through natural consumption in diet and drinking water; however, contributions via cosmetics or industrial activity have also been described [29,30]. In our cohort of patients, barium appears to be correlated with the race/ethnicity of the patients (Table S1). Due to the nonlinear nature of these associations, this observation should be extrapolated with caution, as the vast majority of our population had the same ethnic background (Caucasian). This study suggests that elevated barium levels in follicular fluid are associated with diminished response to ovarian stimulation and compromised embryo development, while urinary barium levels are associated with poor reproductive outcomes and a lower likelihood of achieving a live birth. Consistent with our findings, a study conducted by Jiang and colleagues reported that patients with higher barium concentration in whole blood produced fewer oocytes and had lower fertilization and higher embryo arrest rates [21]. In contrast, another study reported that women exposed to barium produced more mature oocytes and had better blastocyst development. Notably, this group also argued that women overexposed to other toxic elements such as lead and mercury had better reproductive outcomes [24], which are results that should be taken with caution. In this regard, evidence that exposure to high concentrations of barium induces alterations in ovarian morphology, spontaneous abortions, fetal growth restriction, neonatal defects, and death in animal models [30,31] warrants further risk assessment studies in humans.

Strontium is an alkaline metal physiologically present at trace levels—99% of it is sequestered in bone and competes with calcium by forming divalent cations. Strontium may exacerbate kidney disease [32]; however, there is no evidence of dietary toxicity in animal models, and its effects on reproduction and development remain elusive in humans. Strontium chloride is commonly used for artificial oocyte activation because it efficiently triggers waves of intracellular calcium that initiate oocyte activation for parthenogenesis or facilitate embryo fertilization [33,34]. Thus, our study postulated that overexposure to strontium may prematurely activate oocytes, depleting ovarian reserves and dysregulating ovarian function, as has been described for other ovarian toxicants [35]. Our outcomes aligned with this hypothesis, as high strontium concentrations in follicular fluid were negatively associated with AMH levels, oocyte maturation, fertilization, and developmental competence, and plasma strontium negatively affected blastulation and euploidy rates.

Arsenic is a widespread metalloid naturally present in organic and inorganic forms, yet there are few studies exploring its effects on human fertility. We observed that IVF patients with higher plasma arsenic responded poorly to COS and produced fewer mature oocytes and fertilized embryos. Previous studies have reported poorer embryo quality in women exposed to arsenic [23] and lower mean blood concentrations in pregnant women with respect to non-pregnant ones [36], whereas other studies failed to find associations between arsenic exposure and reproductive outcomes [20,21,22,24,26]. In the present study, the total arsenic content was measured, including both the highly toxic inorganic forms and the harmless organic forms—which might lead some to question whether the impact of arsenic was underestimated or overestimated. Nevertheless, recent evidence suggests blood arsenic concentration is a better reflection of exposure to inorganic arsenic than urinary arsenic concentration [37], supporting the reliability of our associations within this matrix. Even so, further studies should be conducted to assess the health risks associated with the different forms of arsenic.

Tin is a malleable metal that can be found in various forms, commonly used in the coating of canned food or beverages and aerosol cans. Inorganic tin compounds are found in cosmetic products, food additives, and dyes, while organic tin, also referred to as organotin compounds, is used to make plastics, food packaging, plastic pipes, pesticides, and paints [38]. Since the current study only assessed the presence of tin ions, different forms of tin were not distinguished between. Our findings linked higher tin levels with lower odds of achieving a live birth within a given IVF cycle, without significantly affecting any of the intermediate variables. To our knowledge, the effect of tin was not previously evaluated with respect to IVF outcomes in humans. However, the Osaka Risk Assessment Division (Japan) found exposure to organotin compounds during the early stages of pregnancy compromised the initiation and maintenance of pregnancy in animal models [39,40,41,42]. The division argued that since reproductive abnormalities were partially due to a decrease in circulating progesterone that hindered decidualization, exogenous progesterone could be used to reverse its effects [39,43,44]. These results reinforce the need for further evaluations of the implications of tin-containing compounds on female fertility.

The present study is among the first to simultaneously analyze barium and tin and propose them as potential risk factors for reproductive success based on the negative associations with live births or obtaining a newborn after a single IVF cycle. Indeed, we demonstrated urinary barium and urinary tin levels were strongly correlated with each other in our cohort of women undergoing IVF, supporting that either metal could be used as a non-invasive biomarker to identify patients with lower odds of achieving their reproductive goals. The correlation of these two metals in urine might reflect common sources of exposures, including dietary (via plants containing high barium from soil contamination or beverages in tin cans) and/or occupational routes [29]. The major strengths of our study included its prospective design that could simultaneously analyze ten non-essential trace elements in three different biological matrices and the stringent inclusion of IVF patients who underwent similar COS protocols followed by ICSI, PGT-A, and SET/FET. This robust design eliminated possible technical and clinical biases associated with laboratory procedures and variations in IVF treatments, respectively. Furthermore, PGT-A ensured the transfer of a euploid embryo, avoiding bias from the developmental potential of the embryo. Finally, delaying SET/FET reduces the serum E2 levels and, consequently, the risks of OHSS and other potential adverse effects on endometrial function. On the other hand, paternal exposure was outside the scope of the current pilot study, which broadly focused on the clinical implications of exposure to non-essential trace elements on endometrial function. While the outcomes of this study evidenced that maternal exposure to certain trace metals is a risk factor associated with poor reproductive outcomes, our findings should be validated in a larger cohort of patients stratified by the different degrees and sources of exposure to these elements. 

## 4. Materials and Methods

### 4.1. Ethical Approval

This prospective, single-center pilot study was approved by the IVI Valencia ethics committee and the Western Institutional Review Board (#1606-FIVI-050-FD). All participants provided written informed consent.

### 4.2. Study Population

In total, sixty women (aged 18–42, with a body mass index (BMI) between 18.6 and 29.9) undergoing IVF following posterior genetic testing for aneuploidy (PGT-A) and single euploid embryo transference (SET/FET) between 2018 and 2019 at RMANJ, Basking Ridge, were included. Women were excluded for severe male-factor infertility, a known history of endometrial insufficiency (i.e., abnormal or unrepaired uterus, irregular endometrium, or endometrial thickness <7 mm on the day of embryo transfer), altered karyotypes, thrombophilia, and endocrinopathies.

### 4.3. Collection of Urine, Blood, and Follicular Fluid Samples

Participants provided a urine and blood sample on the day of vaginal oocyte retrieval (VOR). Urine samples were collected the morning of VOR, under fasting conditions, in sterile polypropylene containers and maintained at 4 °C until centrifugation (500× *g* for 7 min, room temperature). Blood samples were collected in EDTA tubes to isolate plasma by centrifugation (1300× *g* for 15 min, 4 °C). Follicular fluid collected during VOR was centrifuged (1000× *g* for 3 min, room temperature) to remove cellular debris (after isolating the oocytes for clinical use). The urine supernatant, plasma, and follicular fluid samples were all aliquoted and stored at −80 °C until shipment to the IVI Foundation (Valencia, Spain) on dry ice and analysis at the Mass Spectrometry and Geochemical Applications unit of the CIEMAT (Madrid, Spain).

### 4.4. Quantification of Non-Essential Trace Elements

We simultaneously evaluated ten non-essential trace elements (i.e., Ba, Sr, Rb, Sn, Ti, Pb, Cd, Hg, Sb, and As) in the urine, follicular fluid, and plasma samples. Mercury was quantified in unprocessed samples, in triplicate, using a direct mercury analysis system (Tricell DMA-80 instrument, Milestone, Sorisole, Italy) following the recommendations given by the EPA 7473 method [45]. For all other elements, samples were defrosted at 4 °C prior to analysis by Inductively Coupled Plasma Mass Spectrometry (ICP-MS) using an i-CapRQ mass spectrometer (Thermo Fisher Scientific, Madrid, Spain) with a QCell Collision/Reaction Cell (CRC), quadrupole analyzer, and dual-mode secondary electron multiplier (SEM) as a detection system. Urine (0.5 mL) was diluted (1:10) in 2% (*v*/*v*) nitric acid (Sigma-Aldrich). Plasma (0.5 mL) was digested in a DigiPrep block (SCP SCIENCE, Quebec, QC, Canada), with temperature ramping, using 2 mL of 65% nitric acid and 0.1 mL of 40% hydrofluoric acid (75 °C, 15 min) (Merck Millipore, Madrid, Spain) followed by 1 mL of hydrogen peroxide (115 °C, 60 min) (Merck Millipore, Madrid, Spain). Digested samples were supplemented up to 10 mL with Milli-Q water (18.2 MΩ/cm) (Merck Millipore, Madrid, Spain). Finally, follicular fluid samples were diluted (1:20) in 0.5% (*v*/*v*) nitric acid supplemented with 0.0005% (*v*/*v*) Triton^®^ X-100 (Sigma-Aldrich, Madrid, Spain). The internal standards (1 μg/L Ga, In, and Lu) added to the matrices were used as blanks during ICP-MS.

To correct for possible urinary dilutions, we normalized urinary values to the creatinine measured in the aliquoted urine preserved at −80 °C (nanograms of element per gram of creatinine) using the Jaffe reaction measuring the reddish-colored complex resulting from combination of creatinine with alkaline picric acid (Creatinine Parameter Assay Kit, Cat. # KGE005, Bio-Techne R&D Systems, Madrid, Spain). Element concentrations below the level of detection (LOD) were assigned a value equal to half of the LOD.

### 4.5. Clinical Management and Reproductive Outcome Assessment

Participant age, BMI, and baseline AMH were extrapolated from internal electronic medical records, while other demographic variables, such as race/ethnicity, education, and smoking habits, were self-reported in a questionnaire.

A luteal-phase gonadotropin-releasing hormone (GnRH)-antagonist protocol was used for COS. Decisions regarding gonadotrophin doses were based on the clinician’s discretion. When follicles reached 15–20 mm diameter, final oocyte maturation was induced with human chorionic gonadotrophin and/or the GnRH antagonist, and follicles were aspirated by VOR 36 h later.

Oocytes were denudated to distinguish the mature oocytes (metaphase II; MII) from the total recovered. Intracytoplasmic sperm injection (ICSI) was performed in all cases to standardize the fertilization method and reduce the possibility of DNA contamination at the time of PGT-A. Approximately 18 h after ICSI, the number of fertilized oocytes (with two pronuclei) was recorded. Embryos were cultured in sequential culture medium until the blastocyst stage, when embryos underwent a trophectoderm biopsy for PGT-A prior to vitrification.

The SET/FET was performed in a subsequent cycle to ensure embryo–endometrium synchrony. Once the embryo ploidy was confirmed, the participants’ endometria were primed using oral estrogens followed by intramuscular progesterone, as previously described [46]. On the day of the transfer, embryos were thawed and transferred into the endometrial cavity using an ultrasound-guided catheter, according to standard clinical procedures.

Implantation was considered successful when patients presented with a serum β-hCG level > 5 mIU/mL nine days after SET/FET. Clinical pregnancy was confirmed by serum β-hCG ≥ 10 IU/L, along with the presence of an intrauterine gestational sac and/or fetal heartbeat detected by ultrasound within the first 12 weeks of gestation. A live birth was defined as the birth of a live infant after minimum of 24 weeks of gestation.

### 4.6. Statistical Methods

All statistical analyses were performed using R software (version 3.6.2). Participants’ baseline demographic and reproductive characteristics were presented as median ± interquartile ranges (IQR) or absolute counts and proportion of the total (%). The “tableone” package [47] was used to evaluate the associations between non-essential trace element concentrations and baseline demographic and reproductive variables, using Kruskal–Wallis tests for continuous variables and chi-square for categorical variables. The “corrplot” package [48] generated correlation matrices to evaluate the relationships of the distinct non-essential trace elements within and between each biofluid, while the association with IVF outcomes was assessed with generalized linear multivariate mixed models with random intercepts. Gaussian distributions were used to estimate the mean differences in anti-müllerian hormone (AMH) and E2 concentrations, whereas Poisson distributions were employed for discrete count variables (i.e., total recovered oocytes and the relative proportion of MII oocytes, fertilized embryos, blastocysts, and euploid embryos). Non-essential trace element concentrations were modeled as continuous (log-transformed), and linear associations were obtained by comparing the increase between the 20th and 80th percentile. Finally, the “questionr” package [49] generated a binomial distribution to calculate the odds ratios (OR) for clinical IVF outcomes (i.e., implantation, clinical pregnancy, and live birth) relative to the number of SET/FET and reproductive goals (probability of live birth for each cycle).

The multivariate models were adjusted for confounding variables previously related to reproductive outcomes and non-essential trace element exposition [50]. Specifically, the models were adjusted for age (continuous), BMI (continuous), race/ethnicity, and smoking status (never, ever). In all cases, *p* < 0.05 was considered statistically significant.

## 5. Conclusions

In conclusion, the exposure and bioaccumulation of non-essential trace elements such as barium, tin, strontium, and arsenic in different female biological matrices negatively impacted IVF and reproductive outcomes. These data warrant further risk assessments to study the implications of maternal exposure to non-essential trace elements in human reproduction, particularly oocyte maturation and developmental competence, implantation potential, maintenance of pregnancy, and fetal development.

## Figures and Tables

**Table 1:** Baseline demographic and reproductive characteristics.

Demographic Characteristics	
Age (years), median [IQR]	33.40 [31.37, 36.50]
Body mass index (kg/m^2^), median [IQR]	23.87 [21.57, 26.30]
Race/ethnic group, n (%)	
- White/Caucasian	43 (71.7%)
- Afro American	2 (3.3%)
- Asian	6 (10.0%)
- Hispanic	6 (10.0%)
- Other	3 (5.0%)
Post-secondary education, n (%)	55 (94.8%)
Smoking habits, n (%)	
- Never smoked	49 (81.7%)
- Ex-smoker	10 (16.7%)
- Passive smoker	1 (1.7%)
**Reproductive Characteristics**	
Serum anti-müllerian hormone (ng/mL), median [IQR]	3.60 [2.49, 5.17]
Antagonist COS protocol, n (%)	60 (100.0%)
Total FSH dose during stimulation (IU), median [IQR]	2100.00 [1800.00, 2700.00]
Total LH dose during stimulation (IU), median [IQR]	1125.00 [675.00, 1443.75]
Serum estradiol on trigger day (pg/mL), median [IQR]	3750.65 [2622.20, 5204.62]
Number of oocytes recovered, median [IQR]	17.00 [11.00, 24.25]
Number of mature (MII) oocytes, median [IQR]	13.00 [8.75, 20.50]
Oocyte maturation rate, mean ± SD	77.47 ± 14.30%
Number of fertilized embryos (2PN), median [IQR]	11.00 [7.00, 15.25]
Fertilization rate, mean ± SD	81.44 ± 16.29%
Number of blastocysts, median [IQR]	6.00 [3.50, 10.50]
Blastulation rate, mean ± SD	55.62 ± 21.47%
Number of euploid embryos, median [IQR]	3.00 [2.00, 7.00]
Euploidy rate, mean ± SD	60.17 ± 23.72%
Transfer rate, n (%)	55 (91.7%)
Implantation (positive hCG) rate, n (%)	44 (80.0%)
Clinical pregnancy rate, n (%)	38 (69.1%)
Live births, n (%)	35 (63.6%)
Reproductive goal, n (%)	35 (58.3%)

Note: oocyte maturation rate was calculated using the number of MII oocytes over the number of recovered oocytes; fertilization rate was calculated using the number of fertilized embryos over the number of mature oocytes; blastulation rate was calculated using the number of blastocysts over the number of fertilized embryos; and euploidy rate was calculated as the number of euploid blastocysts over the total number of blastocysts obtained. Implantation rate was calculated using the number of patients with positive implantation (serum β-hCG level > 5 mIU/mL nine days after SET/FET) over the total number of patients undergoing embryo transfer. Clinical pregnancy rate was calculated using the number of patients reporting serum β-hCG ≥ 10 IU/L along with the presence of an intrauterine gestational sac and/or fetal heartbeat detected by ultrasound within the first 12 weeks of gestation over the patients reporting positive implantation. The live births indicate the number of neonates delivered following single frozen embryo transfer (SET/FET), whereas the reproductive goal refers to the live births per initiated IVF cycle. IQR, interquartile range; n, number; COS, controlled ovarian stimulation; FSH, follicle-stimulating hormone; LH, luteinizing hormone; IU, international units; MII, metaphase II; SD, Standard deviation; 2PN: two pronuclei; and hCG, human chorionic gonadotropin.

**Table 2:** Non-essential trace element concentrations in follicular fluid, plasma, and urine measured by Inductively Coupled Plasma Mass Spectrometry (ICP-MS).

	Number of Available Samples for Analysis	LOD	Percentage of Samples below LOD (<LOD)	GM (SD)
Follicular Fluid (ng/mL)				
Barium (Ba)	51	2	3.9	2.082 (1.907)
Strontium (Sr)	52	1	0	27.38 (7.595)
Rubidium (Rb)	52	1	0	116.28 (22.915)
Arsenic (As)	52	1	84.6	0.638 (1.271)
Tin (Sn)	52	1	100	
Cesium (Cs)	52	1	100	
Mercury (Hg)	54	0.05	0	1.841 (2.567)
Titanium (Ti)	52	1	0	2.636 (0.759)
Lead (Pb)	48	1	93.8	0.542 (0.361)
Antimonium (Sb)	52	1	100	
Plasma (ng/mL)				
Barium (Ba)	59	1	0	26.24 (121.34)
Strontium (Sr)	59	1	0	37.99 (10.05)
Rubidium (Rb)	59	1	0	361.6 (182.19)
Arsenic (As)	59	2	11.9	4.16 (1.19)
Tin (Sn)	59	5	0	9.55 (5.96)
Cesium (Cs)	59	1	0	3.71 (7.53)
Mercury (Hg)	17	0.5	0	1.8 (1.17)
Titanium (Ti)	59	1	0	567.6 (386.27)
Lead (Pb)	59	1	0	4.3 (1.87)
Antimonium (Sb)	59	5	5.1	9.48 (6.09)
Urine				
Barium (Ba) (ng/mL)	58	1	5.2	1.4 (2.69)
*CR corrected* (µg/g CR)				0.016 (0.021)
Strontium (Sr) (ng/mL)	58	1	0	84.16 (84.6)
*CR corrected* (µg/g CR)				0.966 (0.895)
Rubidium (Rb) (ng/mL)	57	1	0	1125.99 (810.16)
*CR corrected* (µg/g CR)				13.072 (10.843)
Arsenic (As) (ng/mL)	58	0.5	1.7	13.36 (61.02)
*CR corrected* (µg/g CR)				0.153 (0.595)
Tin (Sn) (ng/mL)	58	0.5	15.5	0.92 (1.13)
*CR corrected* (µg/g CR)				0.011 (0.019)
Cesium (Cs) (ng/mL)	58	0.2	0	5.54 (3.84)
*CR corrected* (µg/g CR)				0.064 (0.045)
Mercury (Hg) (ng/mL)	57	0.1	0	1.15 (0.8)
*CR corrected* (µg/g CR)				0.013 (0.029)
Titanium (Ti) (ng/mL)	15	1	0	10.23 (7.34)
*CR corrected* (µg/g CR)				0.121 (0.115)
Lead (Pb) (ng/mL)	58	0.5	34.5	0.46 (0.74)
*CR corrected* (µg/g CR)				0.005 (0.014)
Antimonium (Sb) (ng/mL)	58	1	100	

This table presents the level of detection (LOD) for each element, along with the proportions of undetectable (<LOD) and detectable (>LOD) samples, the geometric mean (GM), and standard deviation (SD). In cases where more than 50% of the quantifications were found to be below the LOD, the element was not assessed. Urinary concentrations were corrected by creatine (CR) levels to account for urine dilution. Follicular fluid, urine, and plasma non-essential trace element concentrations are expressed in ng/mL. Creatinine-corrected values are expressed as µg/g of creatinine (µg/g CR).

**Table 3:** Mean differences (95% CI) for ovarian response and preimplantation IVF outcomes by element concentration.

	Anti-Müllerian Hormone		Trigger Day Estradiol		Number of Retrieved Oocytes		Number of Mature Oocytes		Number of Fertilized Embryos		Number of Blastocysts		Number of Euploid Embryos	
	p20 vs. p80 (95%CI)	*p* Trend	p20 vs. p80 (95%CI)	*p* Trend	p20 vs. p80 (95%CI)	*p* Trend	p20 vs. p80 (95%CI)	*p* Trend	p20 vs. p80 (95%CI)	*p* Trend	p20 vs. p80 (95%CI)	*p* Trend	p20 vs. p80 (95%CI)	*p* Trend
**Follicular Fluid**														
Barium (Ba)	0.59 (0.27, 1.30)	0.86	0.83 (0.70, 0.97)	* 0.019	0.81 (0.66, 0.99)	* 0.042	0.81 (0.66, 0.99)	* 0.041	0.79 (0.64, 0.99)	* 0.038	0.78 (0.63, 0.97)	* 0.027	0.73 (0.60, 0.90)	** 0.004
Strontium (Sr)	0.41 (0.15, 1.14)	0.085	0.88 (0.71, 1.10)	0.251	0.83 (0.65, 1.05)	0.123	0.81 (0.65, 1.01)	0.062	0.81 (0.65, 1.01)	0.06	0.76 (0.62, 0.94)	* 0.014	0.77 (0.63, 0.93)	** 0.008
**Plasma**														
Strontium (Sr)	0.37 (0.15, 0.91)	* 0.032	0.85 (0.70, 1.04)	0.113	0.82 (0.64, 1.03)	0.088	0.77 (0.62, 0.96)	* 0.022	0.77 (0.61, 0.98)	* 0.035	0.77 (0.60, 0.98)	0.036	0.80 (0.62, 1.03)	0.087
Rubidium (Rb)	0.25 (0.08, 0.81)	* 0.022	0.88 (0.68, 1.13)	0.310	0.74 (0.55, 1.01)	0.055	0.78 (0.58, 1.03)	0.083	0.83 (0.61, 1.12)	0.213	0.89 (0.65, 1.22)	0.453	0.94 (0.69, 1.28)	0.69
Arsenic (As)	0.52 (0.21, 1.33)	0.17	0.93 (0.76, 1.13)	0.451	0.74 (0.59, 0.92)	** 0.009	0.75 (0.61, 0.92)	** 0.008	0.77 (0.62, 0.97)	* 0.028	0.83 (0.65, 1.05)	0.116	0.80 (0.63, 1.01)	0.065

Multivariate models were adjusted for age (continuous), BMI (continuous), race/ethnicity, and smoking habits (never, ever). The 20th and 80th percentiles of element distributions for follicular fluid were, respectively, as follows: Ba (1.50 and 2.80), Sr (22.03 and 34.21), Rb (103.20 and 134.00), As (0.50 and 0.50), Ti (2.12 and 3.10), Hg (1.19 and 2.98), and Pb (0.50 and 0.50). The 20th and 80th percentiles of element distributions for plasma were, respectively, as follows: Ba (19.99 and 30.09), Sr (31.01 and 45.39), Rb (264.71 and 578.41), As (3.43 and 5.29), Sn (6.57 and 14.81), Cs (2.45 and 3.93), Ti (328.86 and 908.26), Hg (1.01 and 2.84), Pb (3.03 and 5.65), and Sb (6.46 and 14.11). * *p* < 0.05; ** *p* < 0.01.

## Data Availability

All data presented refer to the original data generated during the study.

## Supplementary Materials

The following supporting information can be downloaded at https://www.mdpi.com/article/10.3390/ijms241713147/s1.
