# Supplementary material for: Bioaccumulation of Non-Essential Trace Elements Detected in Women’s Follicular Fluid, Urine, and Plasma Is Associated with Poor Reproductive Outcomes following Single Euploid Embryo Transfer: A Pilot Study
Source: Int J Mol Sci. 2023 Aug 24;24(17):13147. doi: 10.3390/ijms241713147 (PMC10487767; doi:10.3390/ijms241713147)
Supplement: Supplementary file 1 [file ijms-24-13147-s001.zip › Table S2_Non-essential trace metal concentrations.pdf]

Supplementary Table S2. Non-essential trace metal concentrations in follicular fluid, plasma, and urine measured by Inductively Coupled Plasma Mass Spectrometry (ICP-MS). Supplementary data from Main Table 2.

	Minimum	25%	50%	75%	Maximum
Follicular Fluid (ng/mL)					
Barium (Ba)	0.50	1.50	2.00	2.70	11.10
Strontium (Sr)	9.00	22.27	28.79	32.60	45.00
Rubidium (Rb)	46.00	108.50	124.00	130.25	171.00
Arsenic (As)	0.50	0.50	0.50	0.50	7.08
Tin (Sn)					
Cesium (Cs)					
Mercury (Hg)	0.10	1.22	1.76	2.81	14.90
Titanium (Ti)	1.40	2.20	2.55	3.03	6.10
Lead (Pb)	0.50	0.50	0.50	0.50	2.37
Antimonium (Sb)					
Plasma (ng/mL)					
Barium (Ba)	15.97	20.70	22.86	26.65	953.79
Strontium (Sr)	22.90	32.03	38.30	44.41	73.36
Rubidium (Rb)	194.28	268.64	306.64	488.42	959.40
Arsenic (As)	2.50	3.61	4.11	5.20	8.22
Tin (Sn)	5.25	6.67	8.07	14.22	33.21
Cesium (Cs)	1.97	2.47	3.03	3.62	53.16
Mercury (Hg)	0.59	1.08	1.67	2.80	5.00
Titanium (Ti)	94.24	426.40	618.05	887.93	1697.53
Lead (Pb)	2.58	3.24	4.20	5.22	11.14
Antimonium (Sb)	2.50	6.80	9.63	13.56	38.56

Urine					
Barium (Ba) (ng/mL)	0.25	0.81	1.31	2.47	19.99
CR corrected (µg/g CR)	0.001	0.010	0.019	0.031	0.102
Strontium (Sr) (ng/mL)	9.56	49.28	88.56	150.43	477.11
CR corrected (µg/g CR)	0.103	0.590	1.092	1.519	3.996
Rubidium (Rb) (ng/mL)	110.84	807.99	1161.52	2039.44	3515.73
CR corrected (µg/g CR)	5.002	9.605	12.028	17.490	57.879
Arsenic (As) (ng/mL)	0.25	5.94	12.59	28.29	345.52
CR corrected (µg/g CR)	0.009	0.066	0.126	0.303	3.709
Tin (Sn) (ng/mL)	0.25	0.50	0.92	1.59	5.75
CR corrected (µg/g CR)	0.001	0.006	0.011	0.018	0.108
Cesium (Cs) (ng/mL)	0.58	4.04	5.94	10.13	15.97
CR corrected (µg/g CR)	0.018	0.046	0.062	0.079	0.222
Mercury (Hg) (ng/mL)	0.27	0.78	1.21	1.58	4.94
CR corrected (µg/g CR)	0.003	0.007	0.013	0.023	0.188
Titanium (Ti) (ng/mL)	2.25	6.80	12.07	16.99	27.10
CR corrected (µg/g CR)	0.036	0.068	0.129	0.210	0.491
Lead (Pb) (ng/mL)	0.25	0.25	0.50	0.52	5.80
CR corrected (µg/g CR)	0.001	0.003	0.005	0.010	0.086
Antimonium (Sb) (ng/mL)					

This table presents the distribution of the concentrations among sixty women undergoing IVF with a PGT-A and SET/FET [the minimum, maximum and quartile values (25%, 50%, and 75%) are indicated]. Urinary concentrations were corrected by creatine (CR) levels to account for urine dilution. Follicular fluid, urine and plasma non-essental trace metals concentrations are expressed as ng/mL. Creatinine corrected values are expressed as µg/g of creatinine (µg/g CR).
