# Supplementary material for: Bioaccumulation of Non-Essential Trace Elements Detected in Women’s Follicular Fluid, Urine, and Plasma Is Associated with Poor Reproductive Outcomes following Single Euploid Embryo Transfer: A Pilot Study
Source: Int J Mol Sci. 2023 Aug 24;24(17):13147. doi: 10.3390/ijms241713147 (PMC10487767; doi:10.3390/ijms241713147)
Supplement: Supplementary file 1 [file ijms-24-13147-s001.zip › Table S3_Mean differences (95% CI) for ovarian response and preimplantation IVF outcomes by metal concentration.pdf]

**Supplementary Table S3. Mean differences (95% CI) for ovarian response and preimplantation IVF outcomes by metal concentration. Supplementary data from Main Table 3.**

| | **Anti-müllerian hormone** | | **Trigger day Estradiol** | | **Number of retrieved oocytes** | | **Number of mature oocytes** | | **Number of fertilized embryos** | | **Number of blastocysts** | | **Number of euploid embryos** | |
|---|---|---|---|---|---|---|---|---|---|---|---|---|---|---|
| | p20 vs p80 (95%CI) | p trend | p20 vs p80 (95%CI) | p trend | p20 vs p80 (95%CI) | p trend | p20 vs p80 (95%CI) | p trend | p20 vs p80 (95%CI) | p trend | p20 vs p80 (95%CI) | p trend | p20 vs p80 (95%CI) | p trend |
| **Follicular Fluid** | | | | | | | | | | | | | | |
| Barium (Ba) | 0.59 (0.27, 1.30) | 0.86 | 0.83 (0.70, 0.97) | ***0.019** | 0.81 (0.66, 0.99) | ***0.042** | 0.81 (0.66, 0.99) | ***0.041** | 0.79 (0.64, 0.99) | ***0.038** | 0.78 (0.63, 0.97) | ***0.027** | 0.73 (0.60, 0.90) | ****0.004** |
| Strontium (Sr) | 0.41 (0.15, 1.14) | 0.085 | 0.88 (0.71, 1.10) | 0.251 | 0.83 (0.65, 1.05) | 0.123 | 0.81 (0.65, 1.01) | 0.062 | 0.81 (0.65, 1.01) | 0.06 | 0.76 (0.62, 0.94) | ***0.014** | 0.77 (0.63, 0.93) | ****0.008** |
| Rubidium (Rb) | 0.57 (0.27, 1.18) | 0.127 | 1.06 (0.91, 1.24) | 0.447 | 0.98 (0.82, 1.18) | 0.866 | 0.99 (0.83, 1.17) | 0.871 | 1.00 (0.84, 1.20) | 0.991 | 0.95 (0.80, 1.12) | 0.515 | 0.89 (0.78, 1.02) | 0.102 |
| Mercury (Hg) | 1.26 (0.58, 2.77) | 0.553 | 1.03 (0.86, 1.23) | 0.743 | 1.04 (0.85, 1.28) | 0.679 | 1.04 (0.86, 1.26) | 0.675 | 1.02 (0.83, 1.26) | 0.814 | 0.96 (0.79, 1.16) | 0.649 | 0.98 (0.82, 1.18) | 0.838 |
| Titanium (Ti) | 0.97 (0.33, 2.84) | 0.952 | 1.05 (0.84, 1.31) | 0.679 | 1.05 (0.80, 1.37) | 0.733 | 1.00 (0.75, 1.33) | 0.992 | 0.96 (0.71, 1.30) | 0.799 | 0.96 (0.71, 1.31) | 0.811 | 0.99 (0.72, 1.38) | 0.972 |

| Plasma | | | | | | | | | | | | | | |
|---|---|---|---|---|---|---|---|---|---|---|---|---|---|---|
| Barium (Ba) | 1.01 (0.65, 1.57) | 0.976 | 0.97 (0.88, 1.07) | 0.527 | 1.01 (0.91, 1.12) | 0.899 | 1.02 (0.92, 1.12) | 0.762 | 1.02 (0.92, 1.14) | 0.677 | 1.03 (0.92, 1.16) | 0.572 | 1.07 (0.94, 1.21) | 0.309 |
| Strontium (Sr) | 0.37 (0.15, 0.91) | *0.032 | 0.85 (0.70, 1.04) | 0.113 | 0.82 (0.64, 1.03) | 0.088 | 0.77 (0.62, 0.96) | *0.022 | 0.77 (0.61, 0.98) | *0.035 | 0.77 (0.60, 0.98) | 0.036 | 0.80 (0.62, 1.03) | 0.087 |
| Rubidium (Rb) | 0.25 (0.08, 0.81) | *0.022 | 0.88 (0.68, 1.13) | 0.310 | 0.74 (0.55, 1.01) | 0.055 | 0.78 (0.58, 1.03) | 0.083 | 0.83 (0.61, 1.12) | 0.213 | 0.89 (0.65, 1.22) | 0.453 | 0.94 (0.69, 1.28) | 0.69 |
| Arsenic (As) | 0.52 (0.21, 1.33) | 0.17 | 0.93 (0.76, 1.13) | 0.451 | 0.74 (0.59, 0.92) | **0.009 | 0.75 (0.61, 0.92) | **0.008 | 0.77 (0.62, 0.97) | *0.028 | 0.83 (0.65, 1.05) | 0.116 | 0.80 (0.63, 1.01) | 0.065 |
| Tin (Sn) | 2.76 (0.96, 7.94) | 0.059 | 0.94 (0.75, 1.18) | 0.596 | 0.87 (0.66, 1.14) | 0.31 | 0.90 (0.69, 1.16) | 0.400 | 0.92 (0.70, 1.22) | 0.563 | 0.97 (0.73, 1.30) | 0.835 | 1.01 (0.76, 1.33) | 0.948 |
| Cesium (Cs) | 1.21 (0.79, 1.85) | 0.37 | 1.00 (0.91, 1.09) | 0.993 | 0.99 (0.89, 1.11) | 0.91 | 0.99 (0.89, 1.10) | 0.864 | 0.99 (0.88, 1.11) | 0.833 | 0.98 (0.86, 1.11) | 0.698 | 0.94 (0.82, 1.07) | 0.36 |
| Titanium (Ti) | 0.52 (0.20, 1.35) | 0.175 | 1.01 (0.83, 1.24) | 0.894 | 0.95 (0.74, 1.23) | 0.702 | 0.98 (0.77, 1.24) | 0.841 | 1.00 (0.77, 1.30) | 0.992 | 0.98 (0.74, 1.29) | 0.864 | 1.07 (0.82, 1.40) | 0.617 |
| Lead (Pb) | 1.05 (0.38, 2.94) | 0.923 | 0.97 (0.78, 1.20) | 0.750 | 0.79 (0.61, 1.04) | 0.089 | 0.80 (0.62, 1.04) | 0.096 | 0.85 (0.64, 1.11) | 0.228 | 0.78 (0.58, 1.05) | 0.096 | 0.90 (0.65, 1.23) | 0.493 |

| | | | | | | | | | | | | | | |
|---|---|---|---|---|---|---|---|---|---|---|---|---|---|---|
| Antimonium (Sb) | 1.45 (0.59, 3.56) | 0.416 | 0.94 (0.78, 1.14) | 0.536 | 0.85 (0.69, 1.06) | 0.139 | 0.88 (0.72, 1.08) | 0.205 | 0.89 (0.72, 1.11) | 0.294 | 0.90 (0.72, 1.13) | 0.373 | 0.91 (0.73, 1.14) | 0.422 |
| **Urine** | | | | | | | | | | | | | | |
| Barium (Ba) | 0.84 (0.30, 2.36) | 0.735 | 0.93 (0.75, 1.15) | 0.500 | 0.88 (0.69, 1.11) | 0.273 | 0.82 (0.66, 1.02) | 0.069 | 0.80 (0.64, 1.00) | 0.055 | 0.88 (0.69, 1.11) | 0.276 | 0.90 (0.70, 1.16) | 0.406 |
| Strontium (Sr) | 1.11 (0.38, 3.27) | 0.844 | 0.89 (0.72, 1.12) | 0.315 | 0.85 (0.66, 1.09) | 0.194 | 0.85 (0.67, 1.08) | 0.185 | 0.86 (0.67, 1.11) | 0.232 | 0.95 (0.73, 1.23) | 0.674 | 0.95 (0.71, 1.27) | 0.717 |
| Rubidium (Rb) | 1.32 (0.53, 3.30) | 0.543 | 1.06 (0.89, 1.27) | 0.508 | 0.99 (0.79, 1.23) | 0.931 | 1.01 (0.81, 1.26) | 0.918 | 1.00 (0.80, 1.26) | 0.974 | 0.98 (0.78, 1.24) | 0.888 | 0.98 (0.76, 1.26) | 0.856 |
| Arsenic (As) | 2.09 (0.73, 6.03) | 0.168 | 1.18 (0.95, 1.47) | 0.136 | 1.21 (0.95, 1.54) | 0.116 | 1.19 (0.95, 1.49) | 0.122 | 1.16 (0.92, 1.48) | 0.204 | 1.15 (0.91, 1.47) | 0.233 | 1.16 (0.91, 1.50) | 0.227 |
| Tin (Sn) | 0.88 (0.35, 2.23) | 0.792 | 0.86 (0.72, 1.04) | 0.126 | 0.88 (0.70, 1.11) | 0.275 | 0.89 (0.71, 1.12) | 0.315 | 0.85 (0.67, 1.09) | 0.192 | 0.90 (0.70, 1.16) | 0.405 | 1.03 (0.80, 1.33) | 0.802 |
| Cesium (Cs) | 1.06 (0.45, 2.47) | 0.896 | 1.06 (0.89, 1.26) | 0.536 | 0.98 (0.80, 1.20) | 0.849 | 0.97 (0.80, 1.18) | 0.778 | 0.95 (0.78, 1.16) | 0.616 | 0.97 (0.80, 1.19) | 0.798 | 0.95 (0.77, 1.18) | 0.653 |
| Mercury (Hg) | 1.53 (0.53, 4.42) | 0.425 | 0.95 (0.76, 1.18) | 0.623 | 1.02 (0.78, 1.34) | 0.860 | 1.09 (0.84, 1.41) | 0.516 | 1.08 (0.81, 1.42) | 0.601 | 1.02 (0.77, 1.35) | 0.870 | 1.01 (0.75, 1.35) | 0.961 |

| | | | | | | | | | | | | | | |
|---|---|---|---|---|---|---|---|---|---|---|---|---|---|---|
| Lead (Pb) | 1.06 (0.36, 3.07) | 0.917 | 0.95 (0.76, 1.18) | 0.610 | 0.97 (0.74, 1.28) | 0.840 | 1.02 (0.78, 1.33) | 0.885 | 1.03 (0.78, 1.37) | 0.810 | 1.06 (0.80, 1.41) | 0.672 | 1.04 (0.76, 1.42) | 0.805 |
| Cesium (Cs) | 0.61 (0.22, 1.72) | 0.343 | 1.04 (0.87, 1.26) | 0.640 | 0.89 (0.70, 1.12) | 0.304 | 0.82 (0.66, 1.02) | 0.069 | 0.84 (0.66, 1.06) | 0.132 | 0.81 (0.65, 1.02) | 0,073 | 0.84 (0.67, 1.04) | 0.107 |
| Mercury (Hg) | 1.25 (0.48, 3.22) | 0.642 | 1.00 (0.85, 1.19) | 0.969 | 1.13 (0.92, 1.38) | 0.243 | 1.07 (0.88, 1.30) | 0.487 | 1.08 (0.87, 1.34) | 0.471 | 0.96 (0.78, 1.18) | 0.68 | 0.89 (0.73, 1.09) | 0.259 |

*Multivariate models were adjusted for age (continuous), BMI (continuous), race/ethnicity, and smoking habits (never, ever). The 20th and 80th percentiles of metal distributions for follicular fluid were respectively as follows: Ba (1.50 and 2.80), Sr (22.03 and 34.21), Rb (103.20 and 134.00), As (0.50 and 0.50), Ti (2.12 and 3.10), Hg (1.19 and 2.98), Pb (0.50 and 0.50). The 20th and 80th percentiles of metal distributions for plasma were respectively as follows: Ba (19.99 and 30.09), Sr (31.01 and 45.39), Rb (264.71 and 578.41), As (3.43 and 5.29), Sn (6.57 and 14.81), Cs (2.45 and 3.93), Ti (328.86 and 908.26), Hg (1.01 and 2.84), Pb (3.03 and 5.65), Sb (6.46 and 14.11). Percentiles 20th and 80th of metal distributions for urine (CR corrected) were respectively as follows: Ba (0.01 and 0.03), Sr (0.53 and 1.90), Rb (9.24 and 18.85), As (0.06 and 0.38), Sn (0.01 and 0.02), Cs (0.04 and 0.08), Ti (0.06 and 0.21), Hg (0.01 and 0.02), Pb (0.02 and 0.01). *P<0.05, **P<0.01.*
